# Arabidopsis P4-ATPases ALA1 and ALA7 Enhance Resistance to *Verticillium dahliae* via Detoxifying Vd-Toxins

**DOI:** 10.3390/biology14060595

**Published:** 2025-05-23

**Authors:** Fanlong Wang, Mingliang Qiu, Xiaoxia Yao, Jiancong Li, Hui Ren, Mei Su, Jiaohuan Shen, Caiwang Li, Qian Jiang, Zixuan Zhang, Yundi Li, Jiyu Tang, Xianbi Li, Yanhua Fan, Yan Pei

**Affiliations:** 1 College of Agronomy and Biotechnology, Southwest University, Beibei, Chongqing 400715, China; wfl20220116@swu.edu.cn (F.W.); chongmingdeming@163.com (M.Q.); xiaoxiay0909@163.com (X.Y.); lijiancong9768@outlook.com (J.L.); torenhui@163.com (H.R.); cqnky01@163.com (M.S.); shenjiaol@outlook.com (J.S.); 18624493176@163.com (C.L.); shark322@email.swu.edu.cn (Q.J.); tendency@email.swu.edu.cn (Z.Z.); li18300975365@email.swu.edu.cn (Y.L.); tangjiyu447404132@outlook.com (J.T.); lxianbi@swu.edu.cn (X.L.); fyh@swu.edu.cn (Y.F.); 2 Chongqing Key Laboratory of Crop Molecular Improvement, Southwest University, Beibei, Chongqing 400715, China

**Keywords:** Verticillium wilt, *Verticillium dahliae*, Vd-toxins, P4-ATPases, vesicle trafficking, cell detoxification

## Abstract

Background: Verticillium wilt, which is a soil-borne vascular disease, causes serious economic losses worldwide. Various toxins secreted by *V. dahliae* are key factors that lead to wilt symptoms. Methods: The Vd-toxins CIA, indazole, and 3ICD were labeled with fluorescence groups, respectively, to observe the transport pathway. Transcriptome sequencing and qRT-PCR were employed to assess the expression patterns under Vd-toxin treatment. Results: *AtALA1* and *AtALA7* were up-regulated by *V. dahliae* and LC-toxins, and overexpression of either *AtALA1* or *AtALA7* increased Arabidopsis resistance against LC-toxins. Overexpression of *AtALA1* improved the resistance of Arabidopsis to 4MBA, 3ICD, and indazole, while *AtALA7* enhanced resistance to 4MBA, 3ICD, and CIA. *AtALA7*-overexpressing plants showed a stronger capability to transport CIA^FITC^ and 3ICD^5-FAM^ into vacuoles, while *AtALA1*-overexpressing plants accumulated indazole^5-FAM^ and 3ICD^5-FAM^. Aggregation of *AtALA1* and *AtALA7* enhances the resistance of plants to *V. dahliae*. Conclusions: Arabidopsis P4-ATPase genes *AtALA1* and *AtALA7* mediated cell detoxification by transporting different Vd-toxins to vacuoles for degradation, thereby increasing resistance to Verticillium wilt.

## Simple Summary

The phytopathogenic fungus *Verticillium dahliae* secretes diverse mycotoxins that disrupt host cellular processes, including alteration of microtubule cytoskeletons, nucleoli dysfunction, inhibition of sphingolipid synthesis, and disorder of hydrogen peroxide. However, the mechanisms underlying plant cellular detoxification of mycotoxins secreted by *V. dahliae* remain poorly understood. In this study, we showed that two Arabidopsis P4-ATPases, AtALA1 and AtALA7, are involved in detoxifying distinct Vd-toxins; AtALA1 is responsible for the cell detoxification of indazole, 4MBA, and 3ICD, whereas AtALA7 is essential for CIA, 4MBA, and 3ICD. Transcriptional profiling revealed significant upregulation of reactive oxygen species-associated genes in wild-type Arabidopsis in response to CIA, 3ICD, and indazole exposure. AtALA1- and AtALA7-associated vesicles compartmentalize Vd-toxins and transport them into vacuoles, thereby preventing their binding to intracellular targets and protecting host innate immunity. Notably, the expression of *AtALA1* and *AtALA7* simultaneously further enhances plant resistance against *V. dahliae*. This vesicle trafficking-mediated Vd-toxins detoxification mechanism provides a novel strategy to improve plants against various mycotoxin-producing pathogens, offering broad-spectrum applicability in agricultural biotechnology.

## 1. Introduction

*Verticillium dahliae* is a soil-borne plant fungal pathogen, widely distributed worldwide and well known for causing the destructive wilt disease in many plants [1,2,3,4,5,6]. With a variety of host species, diversity of physiological races, and persistence in soil, Verticillium species are one of the most difficult pathogenic fungi to prevent and control [7,8]. *V. dahliae* produces various toxic secondary metabolites, also known as phytotoxins, responsible for major symptoms associated with Verticillium wilt (Vw) diseases. Varieties of *V. dahliae* toxins (Vd-toxins), including high-molecular-weight protein–lipopolysaccharide complexes and low-molecular-weight toxins, were reported [9,10,11,12,13]. It is reported that low-molecular-weight toxins are the key factors resulting in wilt symptoms [9,11,14]. A lipophilic Vd-toxin, cinnamate acetate (CIA), was identified from a *V. dahliae* strain that is pathogenic to olive trees; CIA induces Vw-like symptoms and thus could be used to select Verticillium-tolerant olive trees [11]. Furthermore, five Vd-toxins containing indole-3-carboxaldehyde (3ICD), 4-hydroxybenzoic acid (4HBA), 2-hydroxyphenylacetic acid (2HPA), 4-methylbenzoic acid (4MBA), and indazole were identified from *V. dahliae* strain ACCC36009 [15]. The Vd-toxin sulfacetamide (SFA) was reported to have the ability to induce necrosis and wilting symptoms in cotton [16]. The sphingolipid biosynthesis inhibitor fumonisin B1 (FB1) is a metabolite produced in *V. dahliae* that likely functions as a virulence factor contributing to Vw symptoms in cotton [17]. Nowadays, the molecular mechanisms involved in plant defense responses to Vd-toxins are poorly understood. The endogenous cyclic adenosine monophosphate (cAMP) was reported to defend responses against Vd-toxins by regulating the production of the salicylic acid signal pathway [18]. Vd-toxins also induce alterations in the cytoskeleton of Arabidopsis cell suspension, as well as in hydrogen peroxide (H_2_O_2_) and nitric oxide [19,20,21]. Furthermore, they reported that H2B monoubiquitination regulates the NADPH oxidase RbohD-dependent H_2_O_2_ production and that the PTP-MPK3/6-WRKY pathway plays an important role in the regulation of RbohD-dependent H_2_O_2_ signaling in defense responses to Vd-toxins in Arabidopsis [22]. The detoxification of phytotoxins was used to prevent and control these insurmountable diseases. This strategy avoids hosts from the harm of mycotoxins, thus allowing hosts to maintain their innate immunity against the invasion of pathogenic fungi and increase their resistance to these diseases [23,24,25,26]. However, the detoxifying mechanisms of Vd-toxins are far from understood.

We recently cloned the *BbCRPA* gene from P4-ATPase of *Beauveria bassiana*, which is involved in the detoxification of cyclosporine A (CsA) and tacrolimus (FK506) via a vesicle trafficking pathway that targets the compounds into vacuoles for detoxification [24]. The P4-ATPases establish and maintain the asymmetrical distribution of lipids in biological membranes via specifically translocating lipid substrates to the cytoplasmic leaflet [27,28,29], which involves various developmental and physiological processes, including plant growth and development, and the response to biotic and abiotic stress [30,31,32,33,34]. Arabidopsis plants contain twelve P4-ATPase members (ALA1 to ALA12) [28], which have been reported to function in temperature stress tolerance [35,36,37], disease resistance [34,38,39,40], plant development [31,32,41,42,43], and heavy metal detoxification [44]. We previously identified that Arabidopsis P4-ATPase genes *AtALA7* and *AtALA1* are involved in detoxifying the Vd-toxin CIA and *Fusarium graminearum* toxin deoxynivalenol (DON). DON/CIA was packaged into AtALA1/AtALA7-mediated vesicles at the plasma membrane, then transported to vacuoles for degradation via the “early endosome–late endosome” vesicle trafficking pathway; the expression of *AtALA1* enhances the resistance of Arabidopsis and maize to *F. graminearum*, and expression of *AtALA7* improves the resistance of Arabidopsis and tobacco to *V. dahliae* [40]. In this study, we further explore the roles of AtALA1 and AtALA7 on Vd-toxins and reveal that AtALA1 is involved in detoxifying 4MBA, indazole, and 3ICD, while AtALA7 is responsible for CIA, 4MBA, and 3ICD. Aggregation of *AtALA1* and *AtALA7* further enhances the resistance of plants to *V. dahliae*, demonstrating a key function of P4-ATPases in Vd-toxin detoxification and Vw resistance.

## 2. Materials and Methods

### 2.1. Plant Materials and Growth Conditions

The *Arabidopsis thaliana* T-DNA insertion mutants *ala1* (salk_002106), *ala7* (salk_063917), and transgenic Arabidopsis plants *35S::AtALA1* (A1-15 and -25), *35S::AtALA7* (A7-9 and -12) used in this study were from our previously published study [40].

Arabidopsis seeds (Col-0) were surface-sterilized in 75% alcohol for 15 min, then rinsed three to five times with sterile water, and sown on an MS medium containing the following: 4.43 g/L MS salt (Murashige and Skoog medium, M519, Phytotech, Lenexa, KS, USA), 15 g/L sucrose, and 2.4 g/L gelrite. Plants were incubated at 4 °C for 2 d in the dark and then transferred to a growth chamber at 21 ± 2 °C with a 16 h light/8 h dark photoperiod, and the light intensity was around 130 μE/m^2^/s.

Tobacco *(Nicotiana tabacum cv. Xanthi)* was grown in a greenhouse at 25 °C under an 18 h light/6 h dark photoperiod, and the light intensity was around 130 μE/m^2^/s. *Agrobacterium tumefaciens* strain EHA105 containing *35S::AtALA1* or *35S::AtALA7* was used for genetic transformation in plants. Cotton plants (*cv.* Jimian 14) were grown as previously described [45,46]. Cotton cotyledons were used for Vd-toxin tolerance assays.

### 2.2. Vd-Toxins Extraction and Phytotoxicity Assays

**Figure 1:**
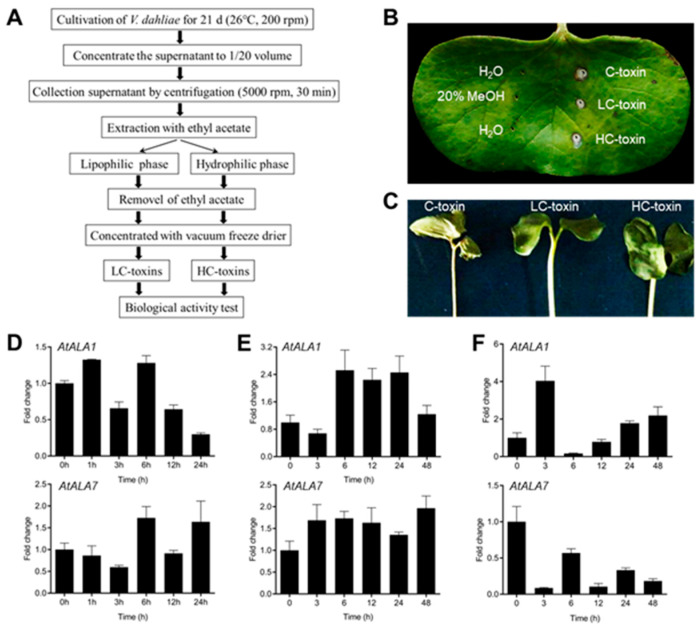
Vd-toxin activity and expression of *AtALA1* and *AtALA7* with different crude toxins of *V. dahliae*. (**A**) Purification process of crude toxins of *V. dahliae* L2-1. (**B**,**C**) Phenotype of cotton cotyledon with different *V. dahliae* toxins (2 μL) for 3 d. C-toxins, crude toxins from *V. dahliae* L2-1 (Czapek liquid medium, 26 °C, 200 rpm for 21 d). LC-toxins, the upper component of C-toxins extracted with ethyl acetate; HC-toxins, the lower component of C-toxins extracted with ethyl acetate. A 20% MeOH was used as a control. (**D**–**F**). The transcription levels of *AtALA1* and *AtALA7* treated with *V. dahliae* strain L2-1 (**D**), LC-toxins (**E**), and HC-toxins (**F**).

The Vd-toxins were extracted from a highly infectious strain of *V. dahliae* L2-1 according to the method shown in Figure 1A. Briefly, the *V. dahliae* strain L2-1 was cultivated in CZM medium at 26 °C and 200 rpm for 21 d. The fungus culture was filtered through four layers of gauze and centrifuged at 10,000 rpm for 10 min to remove the spores. The supernatant was concentrated to 1/20 volume with a vacuum rotary evaporator and centrifuged at 5000 rpm for 10 min. The supernatant after centrifugation (Crude-toxins, C-toxins) was used for the extraction of lipophilic phase crude toxins (LC-toxins) from the lipophilic phase using ethyl acetate, and the extraction of hydrophilic phase crude toxins (HC-toxins) from the hydrophilic phase, respectively. Then, ethyl acetate was removed and concentrated with a vacuum freeze-drier to obtain LC-toxins and HC-toxins.

For phytotoxicity assays of Vd-toxins on cotton cotyledons, 14-day-old cotton cotyledons were pierced into holes with a 1 mL syringe needle, then inoculated with 2 μL of C-toxins, LC-toxins, or HC-toxins, respectively. H_2_O and 20% MeOH were used as controls. After inoculation, these cotyledons were cultured at 26 °C for 3 d with 90% humidity.

For LC-toxin tolerance assays, 2-day-old Arabidopsis plants were transferred to an MS solid medium containing LC-toxins (3 mg·mL^−1^) for 9 d. For CIA tolerance, 4-day-old Arabidopsis plants were transferred to an MS solid medium containing CIA (80 μg·mL^−1^) for 9 d. For Vd-toxin assays in root inhibition, four-day-old plants were transferred to an MS solid medium containing 4MBA (3 μg·mL^−1^), indazole (15 μg·mL^−1^), 2HPA (2 μg·mL^−1^), 4HBA (15 μg·mL^−1^), SFA (20 μg·mL^−1^), or 3ICD (15 μg·mL^−1^) for 7 d, respectively.

### 2.3. Isolation and Quantitative Real-Time PCR (qRT-PCR)

The wild-type Arabidopsis plants grown in MS medium for 14 d were treated with *V. dahliae* L2-1 (2 × 10^8^ spores/mL), LC-toxins (3 mg·mL^−1^), or HC-toxins (3 mg·mL^−1^) for 0, 3, 6, 12, 24, and 48 h. For transcriptome validation, 14-day-old wild-type Arabidopsis plants were treated with CIA (80 μg·mL^−1^), 3ICD (15 μg·mL^−1^), and indazole (15 μg·mL^−1^), respectively. H_2_O was used as a control.

RNA was extracted using the EASY Spin Plant RNA Kit (Aidlab, Beijing, China) according to the manufacturer’s instructions.

CFX Manager 3.1 software (Bio-Rad, Herculus, CA, USA) was used for data analysis. *AtActin2* and *NtActin* were used as reference genes in Arabidopsis and tobacco, respectively. The primers are listed in Table S1.

### 2.4. RNA Sequencing

The 14-day-old wild-type Arabidopsis plants grown on an MS solid medium were transferred to an MS liquid medium or MS containing CIA (80 μg·mL^−1^) for 24 h before RNA isolation. The RNA quality was determined using an Agilent 5300 Bioanalyzer (Agilent, Santa Clara, CA, USA) and quantified using the Nanodrop 2000 (Thermo, Waltham, MA, USA). Only high-quality RNA samples (OD_260/280_ = 1.8~2.2, OD_260/230_ ≥ 2.0, RQN ≥ 6.5, 28S:18S ≥ 1) were used to construct sequencing library.

RNA purification, reverse transcription, library construction, and sequencing were performed at Shanghai Majorbio Bio-pharm Biotechnology Co., Ltd. (Shanghai, China) according to the manufacturer’s instructions. The RNA-seq transcriptome library was prepared following Illumina^®^ Stranded mRNA Prep, Ligation (San Diego, CA, USA) using 1 μg of total RNA. Shortly after, mRNA was isolated according to the polyA selection method by oligo (dT) beads and then fragmented with the fragmentation buffer. Double-stranded cDNA was synthesized with random hexamer primers. The synthesized cDNA was subjected to end-repair, phosphorylation, and adapter addition according to the library construction protocol. Libraries were size-selected for cDNA target fragments of 300–400 bp using magnetic beads, followed by PCR amplification for 10–15 PCR cycles. After being quantified using Qubit 4.0, the sequencing library was performed on the NovaSeq X Plus platform (PE150) using the NovaSeq Reagent Kit (Illumina, San Diego, CA, USA).

To identify DEGs (differentially expressed genes) between two different samples, the expression level of each transcript was calculated according to the transcripts per million reads (TPM) method. Differential expression analysis was performed using DESeq2 or DEGseq. DEGs with |log_2_FC| ≥ 1 and FDR < 0.05(DESeq2) or FDR < 0.001(DEGseq) were considered to be significantly and differentially expressed genes. In addition, functional-enrichment analysis, including GO and KEGG, was performed to identify which DEGs were significantly enriched in GO terms and metabolic pathways at the Bonferroni-corrected *p*-value < 0.05 compared with the whole-transcriptome background. GO functional enrichment and KEGG pathway analysis were carried out on the online platform of Majorbio Cloud Platform (https://cloud.majorbio.com/, accessed on 10 December 2024).

### 2.5. Pathogen Inoculation and Disease Scoring

Arabidopsis and tobacco seedlings were inoculated with *V. dahliae* strains L2-1 (2 × 10^8^ spores/mL) as previously reported [40]. The disease symptoms were classified as level 0–4.0, no visible wilting or chlorosis symptoms; 1, 0–25% (inclusive) of true leaves wilted, chlorosis or dropped off; 2, 25–50% (inclusive) of true leaves wilted, chlorosis or dropped off; 3, 50–75% (inclusive) of true leaves wilted, chlorosis or dropped off; 4, 75–100% (inclusive) of true leaves wilted, chlorosis or dropped off [47,48,49]. The disease index (DI) was obtained according to this formula: DI = [∑ (disease grades × number of infected plants)/(total checked plants × 4)] × 100. A total of 30 Arabidopsis plants and 36 tobacco plants were used for DI analysis.

### 2.6. Laser Confocal Microscopy Observation

Microscopy images were acquired with a Leica SP-8 (Leica Microsystems, Wetzlar, Germany). Images of the Arabidopsis root were acquired with a 40× objective. The pinhole aperture was 1. GFP, CIA^FITC^, 3ICD^5-FAM^, and indazole^5-FAM^ (green channel) were excited at 488 nm, and the emission spectra were 495–540 nm. FM4-64 (red channel) was excited at 552 nm, and the emission spectra were 570–610 nm. The fluorescent intensity in vacuoles was quantified via ImageJ 1.53c (http://imagej.net/ij/, accessed on 16 March 2025). This experiment was independently repeated at least three times.

### 2.7. Statistical Analysis

Statistical analyses were performed with one-way ANOVA with a Tukey’s multiple comparisons test using SPSS (IBM, version 19, Amonk, NY, USA), or Student’s *t*-test using Excel (Microsoft, version 16, Redmond, WA, USA).

## 3. Results

### 3.1. Both AtALA1 and AtALA7 Contribute to the Resistance of LC-Toxins Secreted by V. dahliae in Arabidopsis

The lipophilic phase crude toxins (LC-toxins) and hydrophilic phase crude toxins (HC-toxins) were obtained from a highly infectious strain of *V. dahliae* L2-1, respectively (Figure 1A). Both LC-toxins and HC-toxins showed the ability to induce necrosis and wilting symptoms in cotton cotyledon (Figure 1B,C). We previously identified Arabidopsis P4-ATPase genes *AtALA1* and *AtALA7* as being responsible for the cellular detoxification of mycotoxin DON and CIA, respectively [40]. The transcription level of *AtALA1* and *AtALA7* was induced by treatment of *V. dahliae* strain L2-1 and LC-toxins (Figure 1D,E), and *AtALA1* was also responsive to HC-toxins treatment (Figure 1F).

**Figure 2:**
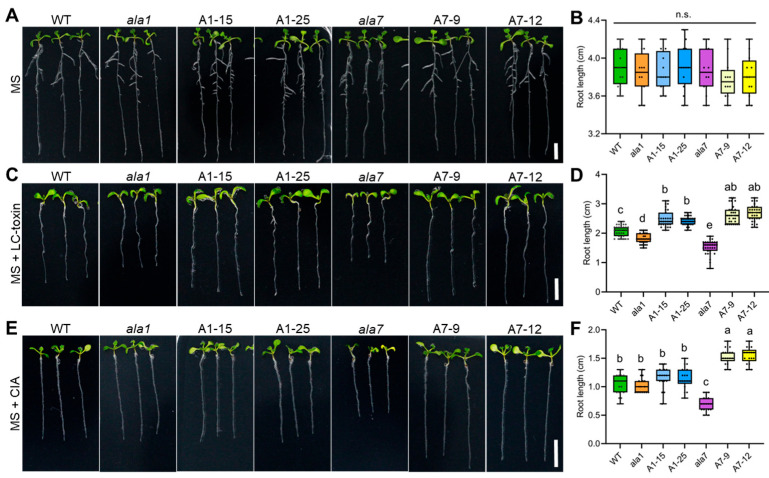
Overexpression of either *AtALA1* or *AtALA7* enhances the resistance of Arabidopsis to LC-toxins. (**A**,**B**) Phenotype and root length of wild-type, *ala1* mutant, *ala7* mutant, *35S::AtALA1* (A1-15, A1-25) and *35S::AtALA7* (A7-9, A7-12) transgenic Arabidopsis plants. These plants were incubated in MS media for 9 d. *ala1*, AtALA1 loss-of-function mutant. *ala7*, AtALA7 loss-of-function mutant. (**C**,**D**) LC-toxin tolerance and root length of wild-type, *ala1* mutant, *ala7* mutant, *35S::AtALA1* and *35S::AtALA7* transgenic Arabidopsis plants. These plants were incubated in MS media containing LC-toxins (3 mg/mL) for 9 d. Scale bar, 0.5 cm. (**E**,**F**) CIA tolerance and root length of wild-type, *ala1* mutant, *ala7* mutant, *35S::AtALA1,* and *35S::AtALA7* transgenic Arabidopsis plants. These plants were incubated in MS solid media containing CIA (80 μg/mL) for 9 d. Different letters in (**B**,**D**,**F**) represent significant differences at *p* < 0.05 by one-way ANOVA with Tukey’s multiple comparisons test. n.s., not significant.

To determine whether AtALA1 and AtALA7 can detoxify LC-toxins, we treated wild-type, *ala1* mutant, *AtALA1* overexpressed Arabidopsis lines (A1-15, A1-25), *ala7* mutant, and *AtALA7* overexpressed Arabidopsis lines (A7-9, A7-12) with LC-toxins and CIA, respectively. There were no significant differences among these plants in the MS medium (Figure 2A,B). In the presence of LC-toxins, *ala1* and *ala7* mutants showed serious toxic symptoms (Figure 2C). The root length of *ala1* and *ala7* mutants was significantly shorter than that of wild-type, while overexpressed lines of *AtALA1* and *AtALA7* were longer than wild-type (Figure 2D). With CIA treatment, the root length of *AtALA7* overexpressed lines was significantly longer than wild-type, and there was no significant difference among wild-type, *ala1* mutant, and *AtALA1* overexpressed lines (Figure 2E,F). These results indicate that overexpression of either *AtALA1* or *AtALA7* can increase Arabidopsis resistance against LC-toxins.

It is reported that *V. dahliae* secreted various secondary metabolites, such as CIA, 3ICD, indazole, 2HPA, 4MBA, and SFA (Table S2). Treated with these Vd-toxins, wild-type Arabidopsis seedlings showed leaf necrosis and death, as well as root growth inhibition (Figure S1). We speculated that these Vd-toxins may play a key role during the colonization of *V. dahliae* into hosts.

### 3.2. Overexpression of Either AtALA1 or AtALA7 Enhances the Resistance of Arabidopsis to LC-Toxins Secreted by V. dahliae

**Figure 3:**
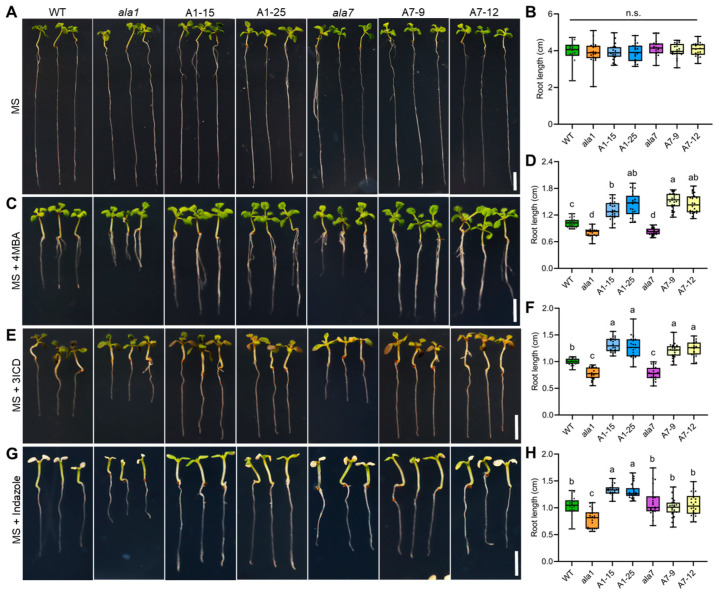
Overexpression of *AtALA1* and *AtALA7* enhances the resistance of Arabidopsis to different Vd-toxins. (**A**,**B**) Phenotype and root length of wild-type, *ala1* mutant, *ala7* mutant, *35S::AtALA1* (A1-15, A1-25) and *35S::AtALA7* (A7-9, A7-12) transgenic Arabidopsis plants. Four-day-old Arabidopsis seedlings were transferred to MS solid media for 7 d. (**C**,**D**) The 4MBA tolerance phenotype and root length of wild-type, *ala1* mutant, *ala7* mutant, *35S::AtALA1* and *35S::AtALA7* transgenic Arabidopsis plants. (**E**,**F**) The 3ICD tolerance phenotype and root length of wild-type, *ala1* mutant, *ala7* mutant, *35S::AtALA1,* and *35S::AtALA7* transgenic Arabidopsis plants. (**G**,**H**) Indazole tolerance phenotype and root length of wild-type, *ala1* mutant, *ala7* mutant, *35S::AtALA1,* and *35S::AtALA7* transgenic Arabidopsis plants. Four-day-old seedlings were transferred to an MS solid medium containing 4MBA (3 μg·mL^−1^), 3ICD (15 μg·mL^−1^), or indazole (15 μg·mL^−1^) for 7 d, respectively. Scale bar, 0.5 cm. Different letters in (**B**,**D**,**F**,**H**) represent significant differences at *p* < 0.05 by one-way ANOVA with a Tukey’s multiple comparisons test. n.s., not significant.

**Figure 4:**
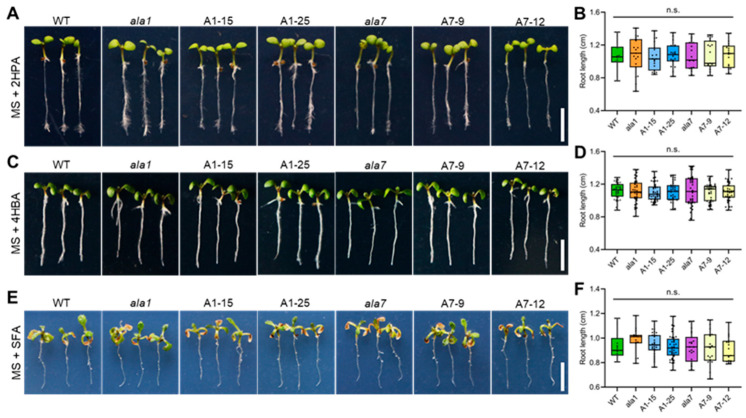
Overexpression of *AtALA1* and *AtALA7* fails to enhance the resistance of Arabidopsis to 2HPA, 4HBA, and SFA. (**A**,**B**) 2HPA tolerance and root length of wild-type, *ala1* mutant, *ala7* mutant, *35S::AtALA1* (A1-15, A1-25) and *35S::AtALA7* (A7-9, A7-12) transgenic Arabidopsis plants. (**C**,**D**) 4HBA tolerance and root length of wild-type, *ala1* mutant, *ala7* mutant, *35S::AtALA1* and *35S::AtALA7* transgenic Arabidopsis plants. (**E**,**F**) SFA tolerance and root length of wild-type, *ala1* mutant, *ala7* mutant, *35S::AtALA1,* and *35S::AtALA7* transgenic Arabidopsis plants. Four-day-old seedlings were transferred to MS solid media containing 2HPA (2 μg·mL^−1^), 4HBA (15 μg·mL^−1^), or SFA (20 μg·mL^−1^) for 7 d. Scale bar, 0.5 cm. n.s., there was no significant difference at *p* < 0.05 by one-way ANOVA with a Tukey’s multiple comparisons test.

To understand whether AtALA1 and AtALA7 can detoxify these Vd-toxins, we treated wild-type, *ala1* mutant, *ala7* mutant, *35S::AtALA1,* and *35S::AtALA7* overexpressed Arabidopsis lines with 4MBA, 3ICD, indazole, 2HPA, 4HBA, and SFA, individually. Treated with 4MBA, the root length of *AtALA1* and *AtALA7* transgenic plants was significantly longer than that of wild-type, while *ala1* and *ala7* mutants were shorter than that of wild-type (Figure 3A–D). Similar to the 4MBA treatment, the transgenic plants of *AtALA1* and *AtALA7* showed less inhibited root growth than wild-type under 3ICD treatment (Figure 3E,F). The root length of *AtALA1* transgenic plants was significantly longer than that of wild-type, and there was no difference among *ala7* mutants, *AtALA7* transgenic plants, and wild-type with indazole treatment (Figure 3G,H). By contrast, overexpression of either *AtALA1* or *AtALA7* did not improve the resistance of Arabidopsis to 2HPA (Figure 4A,B), 4HBA (Figure 4C,D), and SFA (Figure 4E,F). These results showed that the overexpression of *AtALA1* improved the resistance of Arabidopsis to Vd-toxins of 4MBA, 3ICD, and indazole, while overexpressed *AtALA7* enhanced resistance to 4MBA, 3ICD, and CIA.

### 3.3. Overexpression of AtALA1 Promotes Transport Indazole and 3ICD into Vacuoles, While AtALA7 Accumulates CIA and 3ICD to Vacuoles

**Figure 5:**
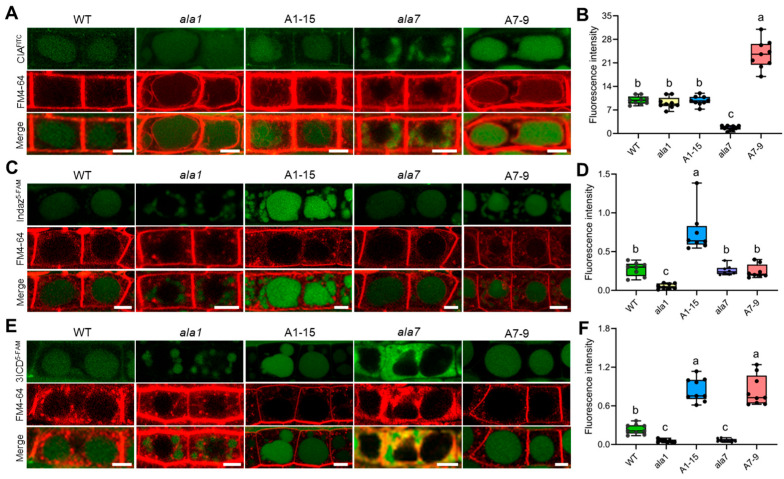
*AtALA7* contributes to the transport of CIA and 3ICD to vacuoles, and *AtALA1* enhances the transport of indazole and 3ICD to vacuoles. (**A**) Distribution of CIA^FITC^ in root cells of wild-type, *ala1* mutant, *ala7* mutant, *35S::AtALA1* (A1-15, A1-25) and *35S::AtALA7* (A7-9, A7-12) transgenic Arabidopsis plants. Two-week-old Arabidopsis seedlings were simultaneously treated with CIA^FITC^ (5 μg·mL^−1^) and FM4-64 (8 μM) for 6 h. CIA^FITC^, CIA was labeled with fluorescein-isothiocyanate-isomer (FITC). Scale bar, 5 μm. (**B**) Fluorescence intensity of CIA^FITC^. (**C**) Distribution of CIA^FITC^ in root cells of wild-type, *ala1* mutant, *ala7* mutant, *35S::AtALA1* and *35S::AtALA7* transgenic Arabidopsis plants. Two-week-old seedlings were treated with indazole^5-FAM^ (30 μg·mL^−1^) and FM4-64 (8 μM) for 8 h. Indazole^5-FAM^, indazole was labeled with 5-carboxyfluorescein (5-FAM). Scale bar, 5 μm. (**D**) Fluorescence intensity of indazole^5-FAM^. (**E**) Distribution of 3ICD^5-FAM^ in root cells of wild-type, *ala1* mutant, *ala7* mutant, *35S::AtALA1,* and *35S::AtALA7* transgenic Arabidopsis plants. Two-week-old seedlings were treated with 3ICD^5-FAM^ (36 μg·mL^−1^) and FM4-64 (8 μM) for 8 h. 3ICD^5-FAM^, 3ICD was labeled with 5-carboxyfluorescein (5-FAM). Scale bar, 5 μm. (**F**) Fluorescence intensity of 3ICD^5-FAM^. Data is shown as dot plots (n = 8 roots). Different letters in (**B**,**D**,**F**) represent significant differences at *p* < 0.05 by one-way ANOVA with a Tukey’s multiple comparisons test.

To investigate the function of AtALA1 and AtALA7 in Vd-toxins detoxification, we treated wild-type, *ala1* mutant, *ala7* mutant, *AtALA1,* and *AtALA7* overexpressed lines with CIA^FITC^ (CIA was labeled with fluorescein-isothiocyanate-isomer), indazole^5-FAM^ (indazole was labeled with 5-carboxyfluorescein), and 3ICD^5-FAM^ (3ICD was labeled with 5-carboxyfluorescein), respectively, which did not impair the toxicity of either indazole (Figure S2A,B) or 3ICD (Figure S2C,D). Similar to our previous result, the transgenic plants overexpressing *AtALA7* exhibited a stronger ability to accumulate CIA^FITC^ into vacuoles than the wild-type, while *AtALA1* was invalid (Figure 5A,B). With indazole^5-FAM^ treatment, the transgenic plants overexpressing *AtALA1* exhibited a more powerful capacity to accumulate indazole^5-FAM^ into vacuoles than the wild-type, while *AtALA7* was ineffective (Figure 5C,D). Interestingly, overexpression of either *AtALA1* or *AtALA7* promotes 3ICD^5-FAM^ accumulation into vacuoles (Figure 5E,F). These results demonstrated that AtALA1 is involved in detoxifying indazole and 3ICD, while AtALA7 is responsible for detoxifying CIA and 3ICD.

### 3.4. AtALA1 or AtALA7 Protect Arabidopsis Plants from ROS Toxicity Triggered by Vd-Toxins

**Figure 6:**
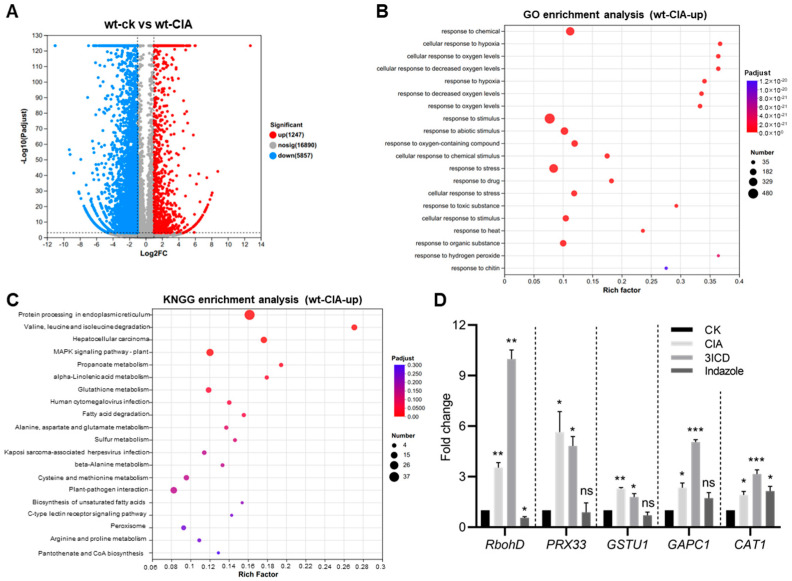
Vd-toxins of CIA, 3ICD, and indazole-induced ROS burst. (**A**) Volcano plots of differentially expressed genes (DEGs) in Arabidopsis wild-type with water containing 0.2% DMSO (wt_ck) or 100 μg·mL^−1^ CIA (wt_CIA). (**B**) GO enrichment upregulated DEGs in wild-type under CIA treatment. (**C**) KNGG enrichment upregulated DEGs in wild-type under CIA treatment. (**D**) Transcript levels of ROS-related genes in wild-type treated with CIA, 3ICD, or indazole. Twelve-day-old wild-type seedlings were transferred to MS media containing CIA (80 μg·mL^−1^), 3ICD (15 μg·mL^−1^), or indazole (15 μg·mL^−1^) for 0.5 h. Data are represented as the mean ± SD. * *p* < 0.05; ** *p* < 0.01; *** *p* < 0.001 from Student’s *t*-test. ns, not significant.

To understand the molecular basis of resistance to Vd-toxins in plants, we analyzed the transcriptomic changes in response to CIA. A total of 7104 DEGs were identified (*p*-adjust ≤ 0.001 and Log2 fold change ≥ 1), including 1247 up-regulated genes and 5857 down-regulated genes (Figure 6A). GO enrichment analysis showed these DEGs are mainly involved in the cellular response to chemical, hypoxia, oxygen levels, stimulus, drug, toxic substance, heat, and hydrogen peroxide (Figure 6B). KNGG analysis showed these DEGs involved in plant–pathogen interactions, MAPK signaling pathways, and peroxisomes (Figure 6C).

We, therefore, analyze the relative expression changes of ROS-related genes (*RbohD*, *PRX33*, *GSTU1*, *CAT1*, and *GAPC1*) in wild-type Arabidopsis under CIA, 3ICD, and indazole treatment, respectively. The qRT-PCR results indicated that all of these selected genes were up-regulated under CIA or 3ICD treatment (Figure 6D); the relative expression of *GSTU1* and *CAT1* was up-regulated, while *RbohD* and *PRX33* were down-regulated under indazole treatment (Figure 6D). These results suggest that Vd-toxins may exert a toxic function by inducing oxidative stress in plant cells.

### 3.5. Aggregation of AtALA1 and AtALA7 Enhances the Plant’s Resistance to V. dahliae

**Figure 7:**
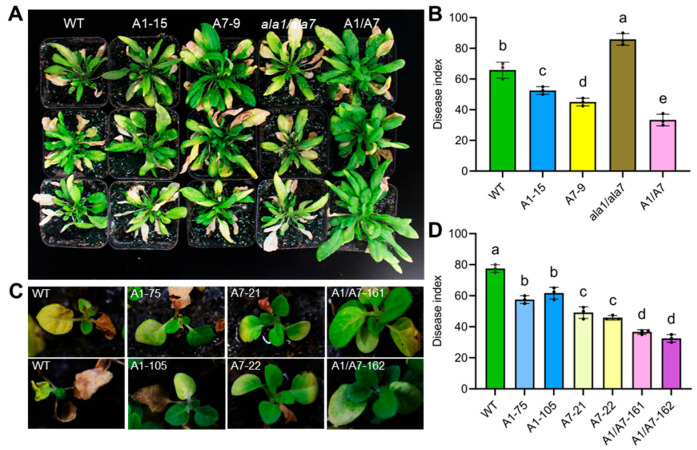
AtALA1 and AtALA7 play key roles in improving Arabidopsis and tobacco resistance to *V. dahliae*. (**A**) Vw symptoms of wild-type, mutant (*ala1/7*), *AtALA1*-overexpressing Arabidopsis lines (A1-15), *AtALA7*-overexpressing Arabidopsis lines (A7-9), and hybrid progeny of *AtALA1* and *AtALA7* (A1/A7). Arabidopsis plants were inoculated with *V. dahliae* (3 mL per plant, 2 × 10^8^ conidia/mL) for three weeks. Scale bar, 2 cm. (**B**) Disease index (DI) at 21 dpi. (**C**) Vw symptoms of wild-type, *AtALA1* transgenic tobacco lines (A1-75 and A1-105), *AtALA7* transgenic tobacco lines (A7-21 and A7-22), and hybrid progeny of *AtALA1* and *AtALA7* (A1/A7-161 and A1/A7-162). Tobacco seedlings were inoculated with *V. dahliae* (10 mL per plant, 2 × 10^8^ conidia/mL) for 3 weeks. Scale bar, 2 cm. (**D**) DI of tobacco plants inoculated with *V. dahliae*. Different letters in (**B**,**D**) represent significant differences at *p* < 0.05 by one-way ANOVA with a Tukey’s multiple comparisons test.

We further analyzed the resistance of *AtALA1* and *AtALA7* transgenic Arabidopsis to a strong pathogenic strain, *V. dahliae* L2-1. After inoculation, the *ala1/ala7* mutant presented the most serious disease symptoms, while the hybrid progeny of *AtALA1* and *AtALA7* (A1/A7) showed fewer symptoms (Figure 7A). The DI of *AtALA1* and *AtALA7* transgenic Arabidopsis was significantly lower than wild-type, while the hybrid progeny (A1/A7) was lower than plants expressing *AtALA1* or *AtALA7* alone (Figure 7B). Similarly, the DI of hybrid tobacco progeny (A1/A7) was lower than plants expressing *AtALA1* or *AtALA7* alone (Figure 7C,D). These data suggest that the expression of *AtALA1* and *AtALA7* simultaneously can further enhance plant resistance against *V. dahliae*.

## 4. Discussion

Due to the broad host range, complicated pathogenic mechanisms, high genetic diversity, and the lack of resistance germplasm resources to Verticillium species, the control of Vw is far from successful [50,51,52]. Many low molecular secondary metabolites (also called mycotoxins) secreted by pathogenic fungi play a crucial role during pathogenesis [9,53,54]. *V. dahliae* belongs to hemibiotrophic pathogens. These pathogens secrete low levels of mycotoxins to suppress the host’s immune response and thus promote their colonization at an early stage of infection, and also generate high levels of mycotoxins to stimulate cell death [55,56]. For example, the trichothecenes family of phytotoxins, mainly produced by necrotrophic fungal phytopathogenic Fusarium species, contains more than 200 members and is divided into four types, and T-2 toxin is the most dangerous mycotoxins [57,58]. The Vd-toxins 3ICD, indazole, 2HPA, 4MBA, and SFA cause leaf necrosis and death, and root growth inhibition in wild-type Arabidopsis seedlings with different concentrations (Figure S1, Figure 3 and Figure 4), implying that Vd-toxins play different roles during the pathogenesis process, which may be related to their structure, sites of action, and targets in plants.

The detoxification of mycotoxins has been developed to control these formidable diseases [59,60,61]. Nowadays, some Vd-toxins have been identified from *V. dahliae* [1,11,12,17,22,62,63], which are involved in inducing Vw-like symptoms [11,53,64], altering microtubule cytoskeletons and nucleoli [19,21], inhibiting sphingolipid synthesis [17], and causing disorder of hydrogen peroxide [22]. However, the studies on improving the cell detoxification function of plants against Vd-toxins are limited. We recently cloned the *BbCRPA* gene from P4-ATPase of *Beauveria bassiana*, which is involved in the detoxification of mycotoxins CsA and FK506 via a vesicle trafficking pathway, and exogenous overexpression of *BbCRPA* enhances the resistance to *V. dahliae* via transport CIA to vacuoles in Arabidopsis [24]. We also identified Arabidopsis P4-ATPase genes *AtALA1* and *AtALA7*, which contribute to cellular detoxification of DON and CIA, respectively, using a classical clathrin-associated endocytosis mechanism [40].

In this study, we demonstrate that AtALA1, a membrane of P4-ATPases, mediates the detoxification of 4MBA, indazole, and 3ICD via vesicle trafficking, whereas AtALA7 facilitates CIA, 4MBA, and 3ICD (Figure 3 and Figure 5). Notably, co-expression of *AtALA1* and *AtALA7* synergistically enhances plant resistance against *V. dahliae* (Figure 7). These low-molecular-weight Vd-toxins exhibit high lipophilicity, as evidenced by their high LogP values (for CIA, 2.62; indazole, 1.82; 4MBA, 2.36; 3ICD, 1.68; Table S2). Thus, we supposed that the cargo mediated by P4 ATPase-associated vesicle trafficking may be a group of molecules with similar physicochemical or biological properties. It is reported that AtALA1 is also involved in chilling tolerance and antiviral RNA interference [35,38,39]. The lipid substrate of AtALA1 was PS (phosphatidylserine), while with AtALA7 is still unclear [28,35,65]. It seems that the P4-ATPases are unlikely to directly participate in compound recognition but rather act as transporters within broader regulatory networks. For instance, mammal P4-ATPase ATP11C undergoes endocytosis mediated by serotonin or histamine binding to G protein-coupled receptors (GPCRs), followed by Ca^2+^ influx and PKCa-dependent phosphorylation of its C-terminus [66]. Recently, our work revealed that the DON-stimulated phosphorylation in the C-terminus is responsible for promoting DON trafficking into vacuoles, and the WNK10 kinase could interact with the C-terminus of AtALA1 [66]. Therefore, indazole, 3ICD, and DON may represent a subset of cargo trafficked by AtALA1-mediated vesicle pathways. Elucidating the upstream signaling components of the plasma membrane/intracellular under Vd-toxin induction, and combining with biochemical assays, may provide a basis for recognizing structurally diverse compounds.

Many mycotoxins were reported to induce oxidative stress, thereby inducing apoptosis [67]. For example, both T-2 and DON-induced apoptosis are related to DNA methylation; DON triggers early-stage apoptosis and autophagy [68,69,70,71]. The ROS could be produced in the plasma membrane (RbohD), peroxisome (PRX33), and mitochondria, while the CAT1 and GSTU1 are responsible for ROS scavenging [72]. The cytosolic glycolytic enzymes glyceraldehyde-3-phosphate dehydrogenases (GAPCs) were reported to interact with the plasma membrane-associated phospholipase D to transduce hydrogen peroxide in Arabidopsis [73]. The expression of *RbohD*, *RbohF,* and *GST1* was upregulated under Vd-toxin treatment [22]. In this study, DEGs under CIA treatment are involved in the cellular response to oxygen levels and hydrogen peroxide (Figure 6B), and ROS-related genes such as *RbohD*, *GSTU1*, *PRX33*, *GAPC1*, and *CAT1* were up-regulated in wild-type Arabidopsis under CIA and 3ICD (Figure 6D), indicating that Vd-toxins, such as CIA and 3ICD, also triggered the response to ROS-related genes in plants. The P4-ATPases AtALA1 and AtALA7 achieve detoxification via transport of Vd-toxins to vacuoles. Once inside cells, Vd-toxins are encapsulated in vesicles, preventing the contact and binding of toxins to their intracellular targets, thus protecting the innate immunity system of hosts from damage and increasing the resistance of hosts against invasion of *V. dahliae*. Ultimately, the Vd-toxins are transported into vacuoles to be sequestered or digested.

## 5. Conclusions

In this study, we investigated the damage of Vd-toxins to plants. Both LC-toxins and HC-toxins showed the ability to induce necrosis and wilting symptoms in cotton cotyledons. Vd-toxins such as CIA, 3ICD, indazole, 2HPA, 4MBA, and SFA showed leaf necrosis and root growth inhibition. Overexpressing *AtALA1* improved the resistance of Arabidopsis to 4MBA, 3ICD, and indazole, while overexpressing *AtALA7* enhanced resistance to 4MBA, 3ICD, and CIA. By observing the distribution of fluorescently labeled Vd-toxins (CIA^FITC^, indazole^5-FAM^, and 3ICD^5-FAM^) in Arabidopsis root cells, we demonstrated that *AtALA7* overexpressing plants exhibited a stronger ability to accumulate CIA^FITC^ into vacuoles than wild-type, while *AtALA1* was invalid; *AtALA1* overexpressing plants showed a stronger accumulate indazole^5-FAM^ into vacuoles than wild-type, while overexpression of either *AtALA1* or *AtALA7* promotes 3ICD^5-FAM^ accumulation into vacuoles, indicating that AtALA1 is involved in detoxifying indazole and 3ICD, while AtALA7 is responsible for cell detoxification CIA and 3ICD. The ROS-related genes, such as *RbohD*, *PRX33*, *GAPC1*, and *CAT1,* were up-regulated under Vd-toxins treatment. Furthermore, the expression of *AtALA1* and *AtALA7* simultaneously further enhances plant resistance against *V. dahliae*.

## Data Availability

The data presented in this study are available on request from the corresponding author. The data are not publicly available due to privacy.

## Supplementary Materials

The following supporting information can be downloaded at https://www.mdpi.com/article/10.3390/biology14060595/s1. Figure S1: Vd-toxins tolerance assay of wild-type Arabidopsis seedling; Figure S2: 5-FAM labeled do not impair the toxicity of indazole and 3ICD to wild-type Arabidopsis. Table S1: Primers used in this study. Table S2: Summary of reported Vd-toxins of *V. dahliae*.

## Abbreviations

The following abbreviations are used in this manuscript:

CIA	Cinnamyl acetate
cAMP	Cyclic adenosine monophosphate
C-toxins	Crude toxin
DGEs	Differentially expressed genes
HC-toxins	Hydrophilic phase crude toxin
H_2_O_2_	Hydrogen peroxide
KNGG	Kyoto encyclopedia of genes and genomes
LC-toxins	Lipophilic phase crude toxin
qRT-PCR	Quantitative real-time polymerase chain reaction
rpm	Revolutions per minute
SFA	Sulfacetamide
Vw	Verticillium wilt
2HPA	2-hydroxypenylacetic acid
3ICD	Indole-3-carboxaldehyde
4HBA	4-hydroxybenzoic acid
4MBA	4-methylbenzoic acid
5-FAM	5-carboxyfluorescein
