# Supplementary material for: Arabidopsis P4-ATPases ALA1 and ALA7 Enhance Resistance to Verticillium dahliae via Detoxifying Vd-Toxins
Source: Biology (Basel). 2025 May 23;14(6):595. doi: 10.3390/biology14060595 (PMC12189932; doi:10.3390/biology14060595)
Supplement: Supplementary file 1 [file biology-14-00595-s001.zip › biology-3626914-supplementary/biology-3626914-supplementary.pdf]

**Arabidopsis P4-ATPases ALA1 and ALA7 enhance resistance to *Verticillium dahliae* via detoxifying Vd-toxins**

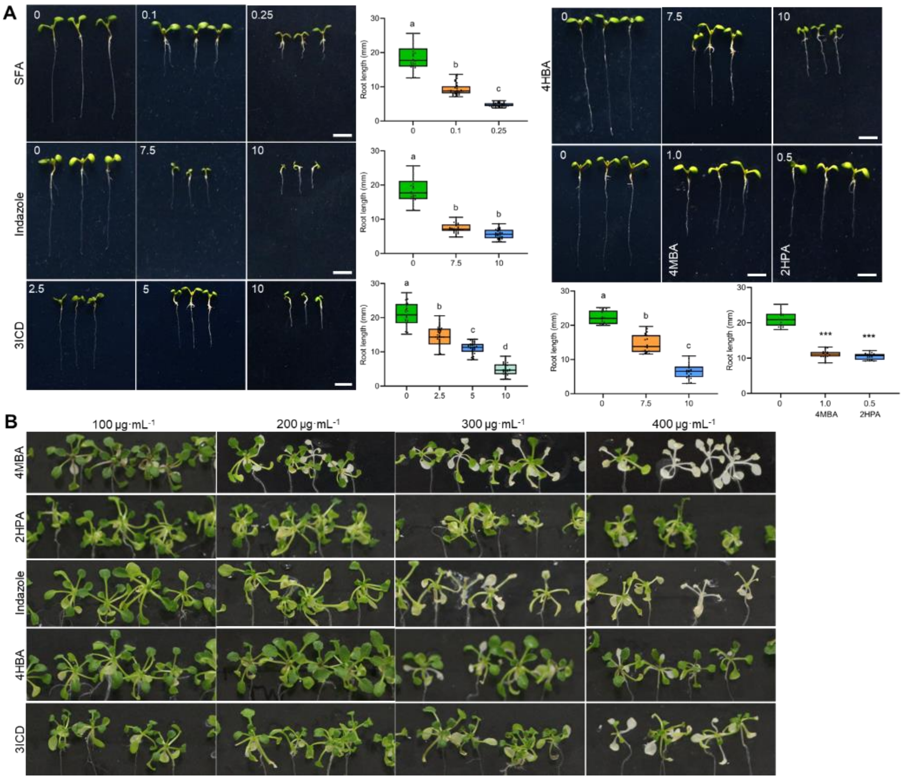


**Figure S1. Vd-toxins tolerance assay of wild-type Arabidopsis seedling.** (**A**) Phenotype and root length of wild-type Arabidopsis seedlings treated with different Vd-toxins. The wild-type Arabidopsis seedlings were exposed to different Vd-toxins for 7 d. SFA (Sulfacetamide) with 0, 0.1 and 0.25 µg·mL$^{-1}$. Indazole with 0, 7.5 and 10 µg·mL$^{-1}$. 3ICD (Indole-3-carboxaldehyde) with 2.5, 5 and 10 µg·mL$^{-1}$. 4HBA (4-hydroxybenzoic acid) with 0, 7.5 and 10 µg·mL$^{-1}$. 4MBA (4-methylbenzoic acid) with 1 µg·mL$^{-1}$. 2HPA (2-hydroxypenylacetic acid) with 0.5 µg·mL$^{-1}$. Scale bar, 0.5 cm. Box-and-whisker plots show the medians (horizontal lines), upper and lower quartiles (box edges), and 1.5× the interquartile range (whiskers). Different letters represent significant differences at $P < 0.05$ by one-way ANOVA with a Tukey multiple comparisons test. Box-and-whisker plots show the medians (horizontal lines), upper and lower quartiles (box edges), and 1.5× the interquartile range (whiskers). A paired one-tailed *t*-test was conducted. ***$p < 0.001$. (**B**) Vd-toxins tolerance assay of wild-type Arabidopsis seedling. Seven-day-old seedlings were exposed to different Vd-toxins with different concentrations (100, 200, 300, and 400 µg·mL$^{-1}$) for 5 d.

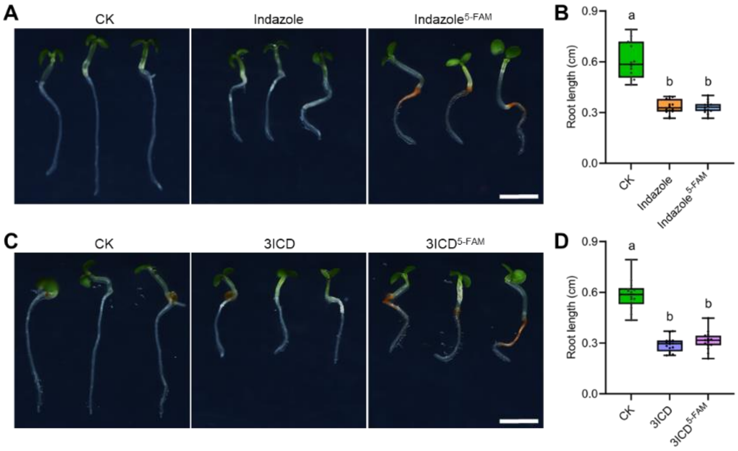


**Fig S2. 5-FAM labeled do not impair the toxicity of Indazole and 3ICD to wild-type Arabidopsis.** (**A**) Phenotype of wild-type Arabidopsis seedlings to Indazole. CK, two-day-old wild-type Arabidopsis seedlings were treated with sterile water for 5 d. Indazole, two-day-old wild-type Arabidopsis seedlings were treated with Indazole (15 μg·mL$^{-1}$) for 5 d. Indazole$^{5\text{-}FAM}$, two-day-old wild-type Arabidopsis seedlings were treated with Indazole$^{5\text{-}FAM}$ (15 μg·mL$^{-1}$) for 5 d. Scale bar, 0.2 cm. (**B**) Root length of wild-type Arabidopsis seedlings treated with Indazole and Indazole$^{5\text{-}FAM}$. (**C**) Phenotype of wild-type Arabidopsis seedlings to 3ICD. CK, two-day-old wild-type Arabidopsis seedlings were treated with sterile water for 5 d. 3ICD, two-day-old wild-type Arabidopsis seedlings were treated with 3ICD (15 μg·mL$^{-1}$) for 5 d. 3ICD$^{5\text{-}FAM}$, two-day-old wild-type Arabidopsis seedlings were treated with 3ICD$^{5\text{-}FAM}$ (15 μg·mL$^{-1}$) for 5 d. Scale bar, 0.2 cm. (**D**) Root length of wild-type Arabidopsis seedlings treated with 3ICD and 3ICD$^{5\text{-}FAM}$. Box-and-whisker plots show the medians (horizontal lines), upper and lower quartiles (box edges), and 1.5× the interquartile range (whiskers). Different letters in (**B**) and (**D**) represent significant differences at $P < 0.05$ by one-way ANOVA with a Tukey multiple comparisons test. Box-and-whisker plots show the medians (horizontal lines), upper and lower quartiles (box edges), and 1.5× the interquartile range (whiskers).
